# Supplementary figures and images for: An inherently interpretable AI model improves screening speed and accuracy for early diabetic retinopathy
Source: PLOS Digit Health. 2025 May 12;4(5):e0000831. doi: 10.1371/journal.pdig.0000831 (PMC12068651; doi:10.1371/journal.pdig.0000831)

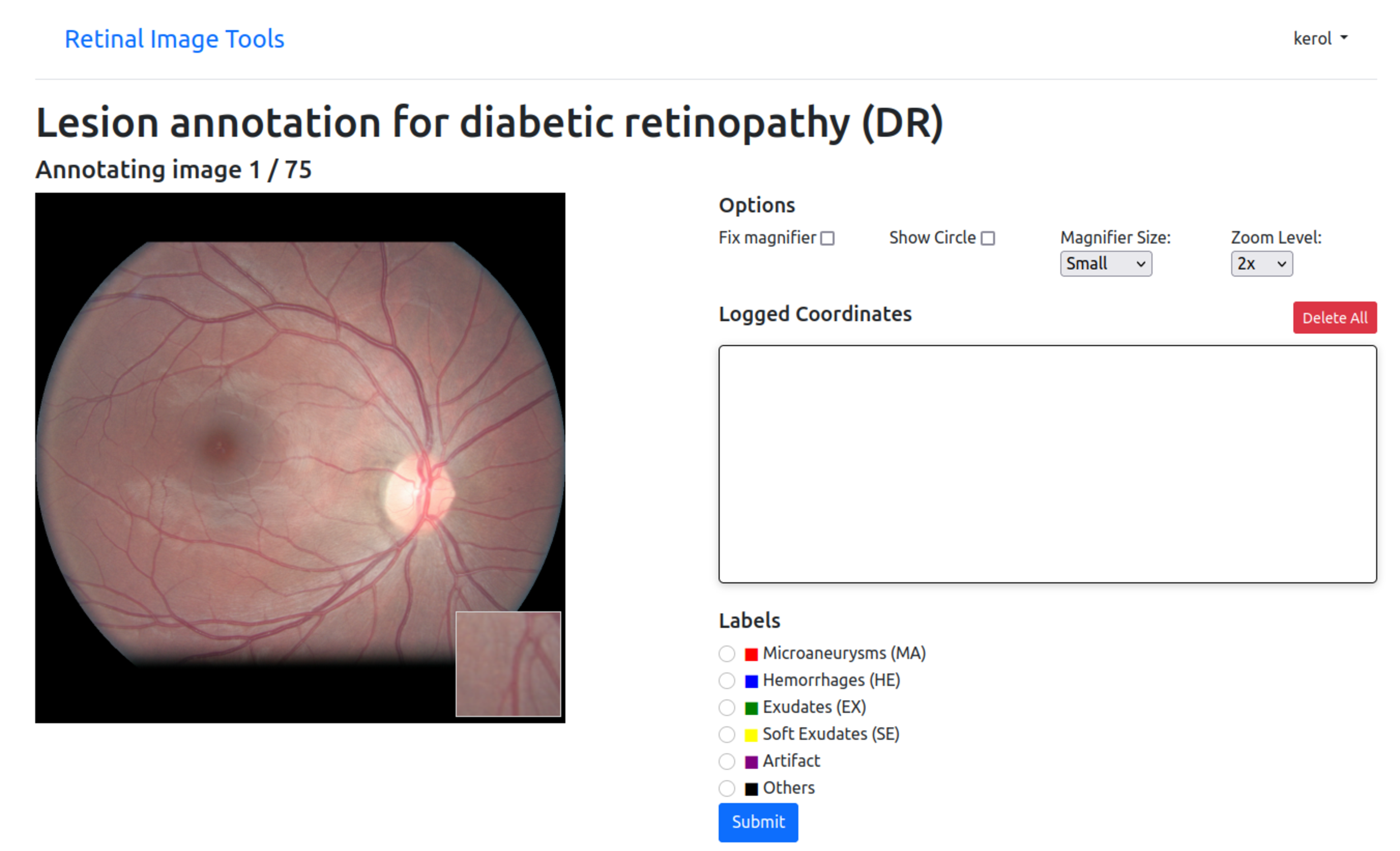

Supplement: S1 Fig — A fundus image is shown and based on it, the annotator is asked to annotate lesions related to Diabetic Retinopathy. By moving the mouse over a region of the image, an enlarged version of that region is displayed. All images are from patients with DR of grade 1 (“mild DR”) or 2 (“moderate DR”). Each lesion is marked by selecting the type (Microaneurysms: MA, hemorrhages: HE, exudates: EX, soft exudate: SE, artifact, or any other lesions) and clicking on the image location. (TIF) [file pdig.0000831.s001.tif]

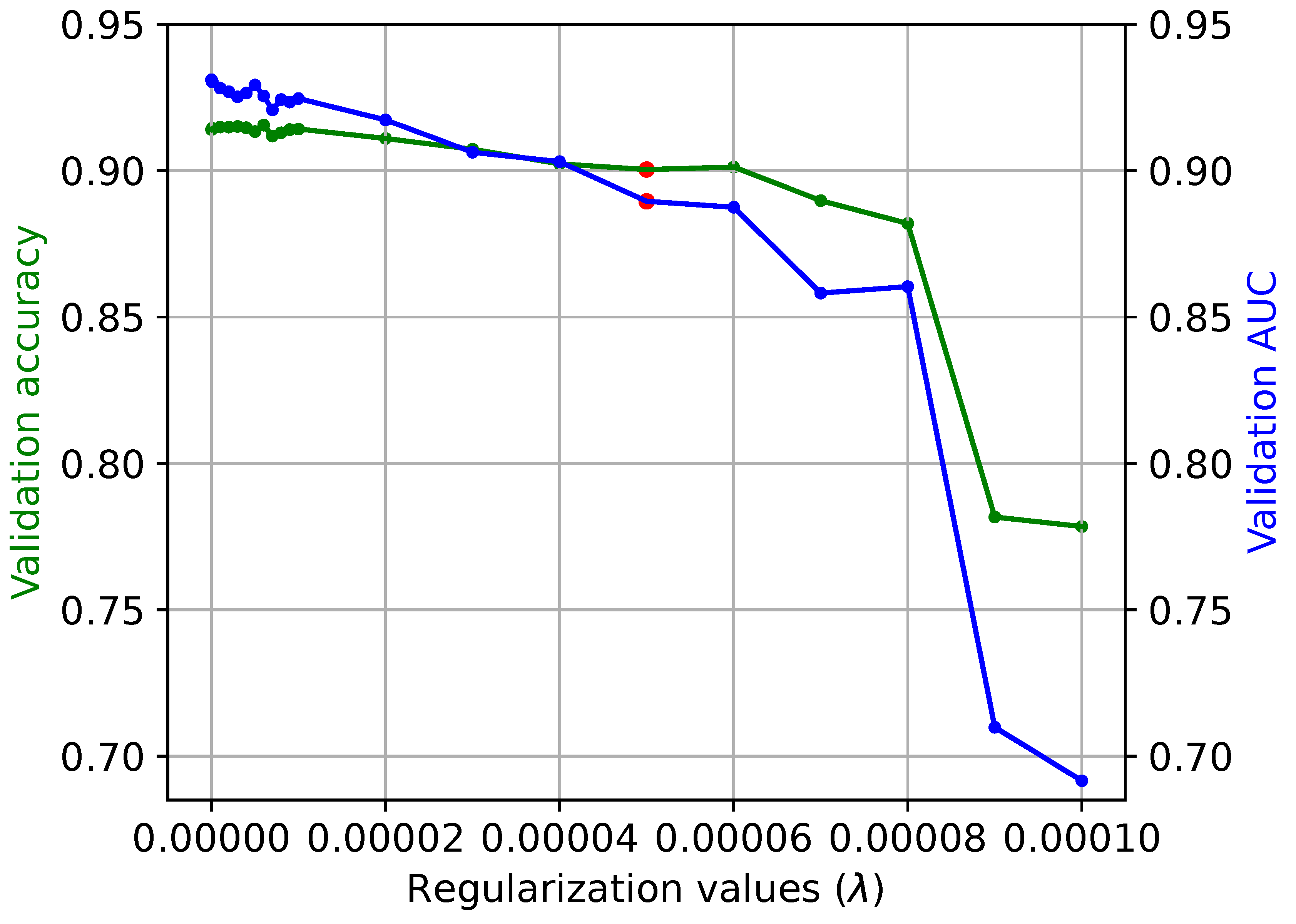

Supplement: S2 Fig — The regularization coefficient λ affects the classification performance (accuracy and AUC) of the model. The red points indicate the selected value, which is a compromise between sparsity and both accuracy and AUC. It also defines the trade-off between the model’s interpretability and classification performance. (TIF) [file pdig.0000831.s004.tif]

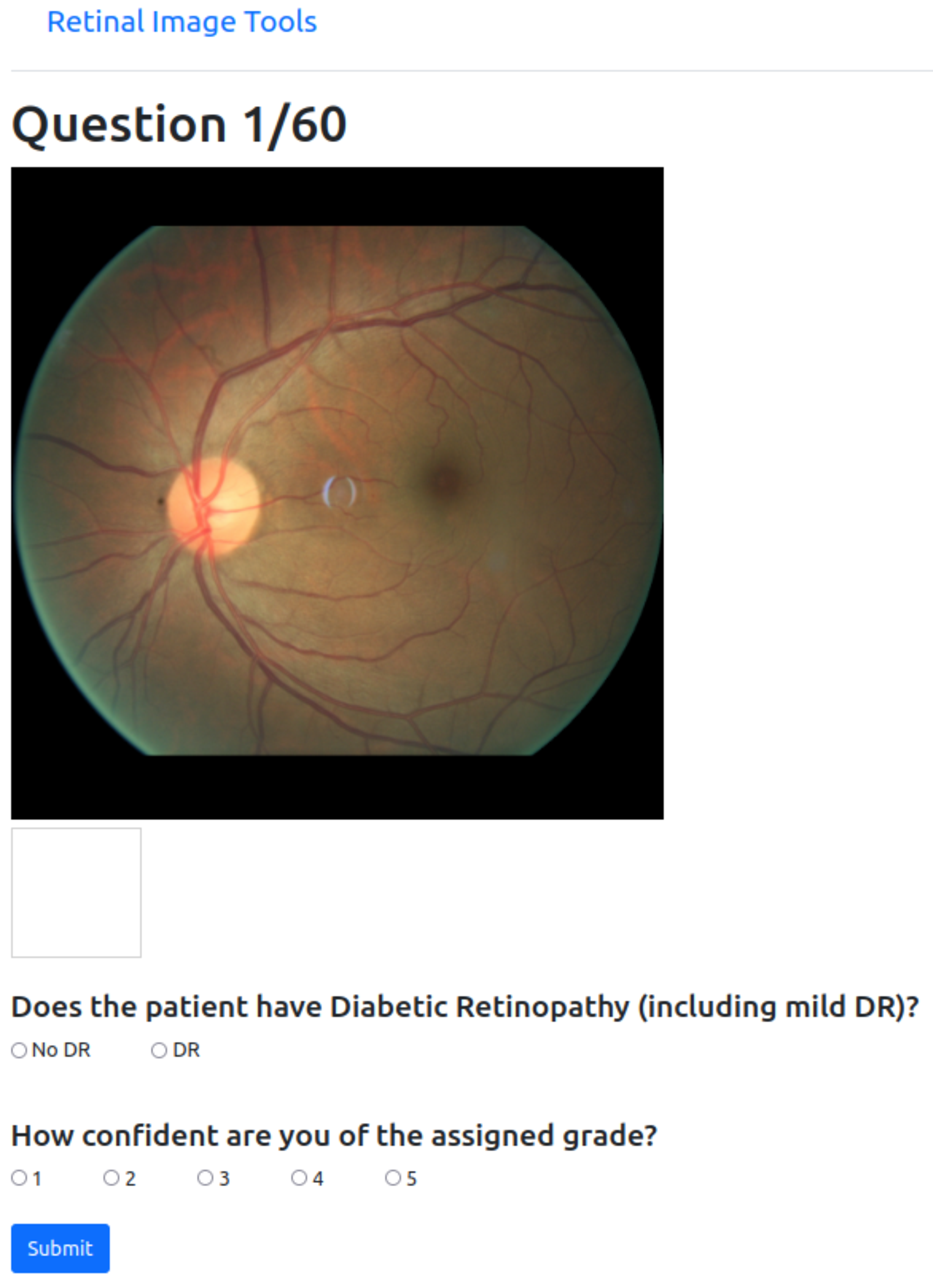

Supplement: S3 Fig — A fundus image is shown and based on it, the grader is asked to decide whether the corresponding patient has Diabetic Retinopathy (DR) of any severity, including mild DR. In addition, the grader is asked to rate the confidence of his/her decision on a scale from 1 (least confident) to 5 (most confident). By moving the mouse over a region of the image, an enlarged version of that region is displayed. The time taken to reach each decision (grading and confidence) is recorded. (TIF) [file pdig.0000831.s005.tif]

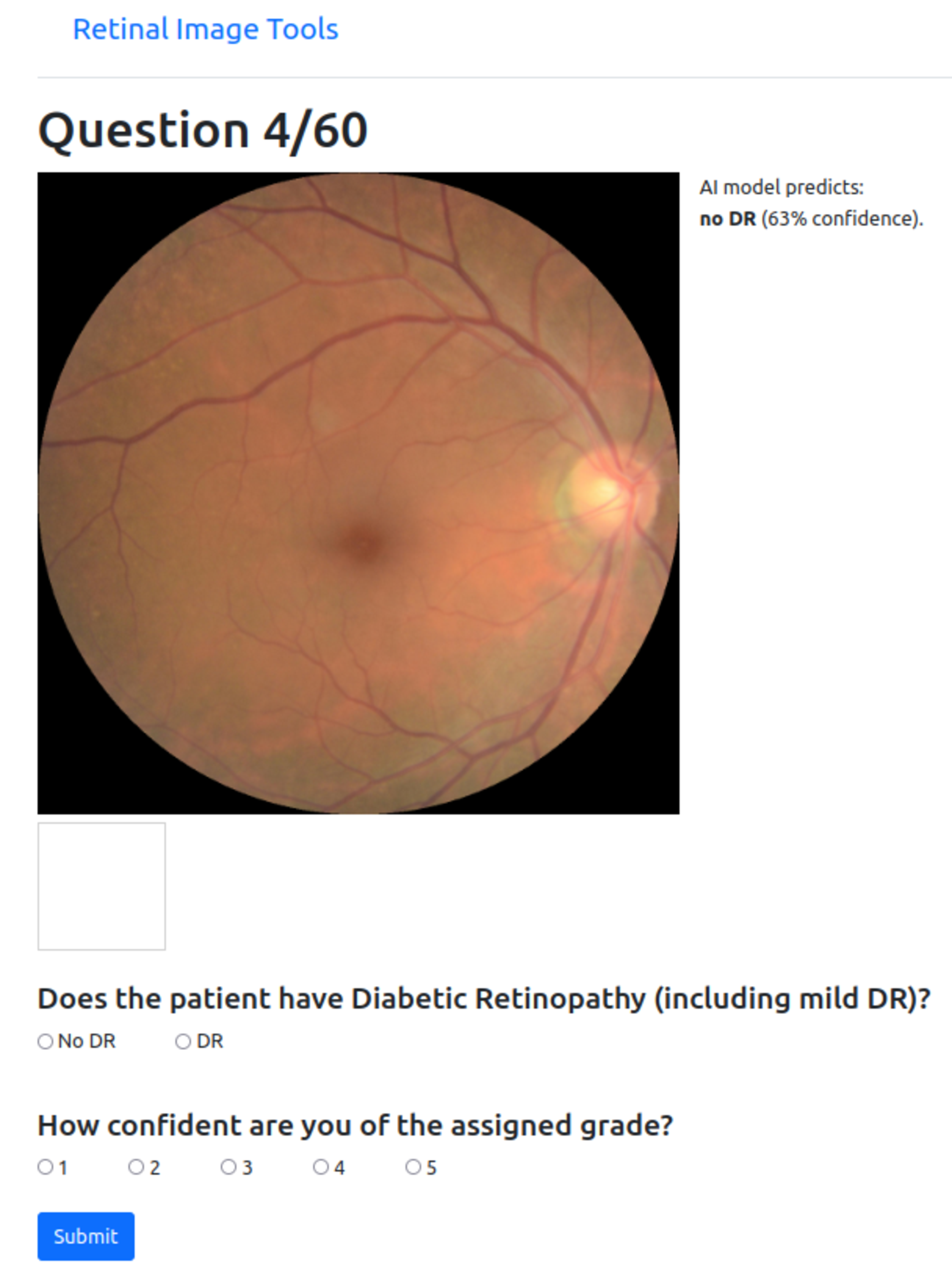

Supplement: S4 Fig — A fundus image is shown with the model’s prediction and its confidence level (from 0% to 100 %, with 100% being the highest confidence score). Based on this, the grader is asked to decide whether the corresponding patient has Diabetic Retinopathy (DR) of any severity, including mild DR. In addition, the grader is asked to rate the confidence of his/her decision on a scale from 1 (least confident) to 5 (most confident). By moving the mouse over a region of the image, an enlarged version of that region is displayed. The time taken to reach each decision (grading and confidence) is recorded. (TIF) [file pdig.0000831.s006.tif]

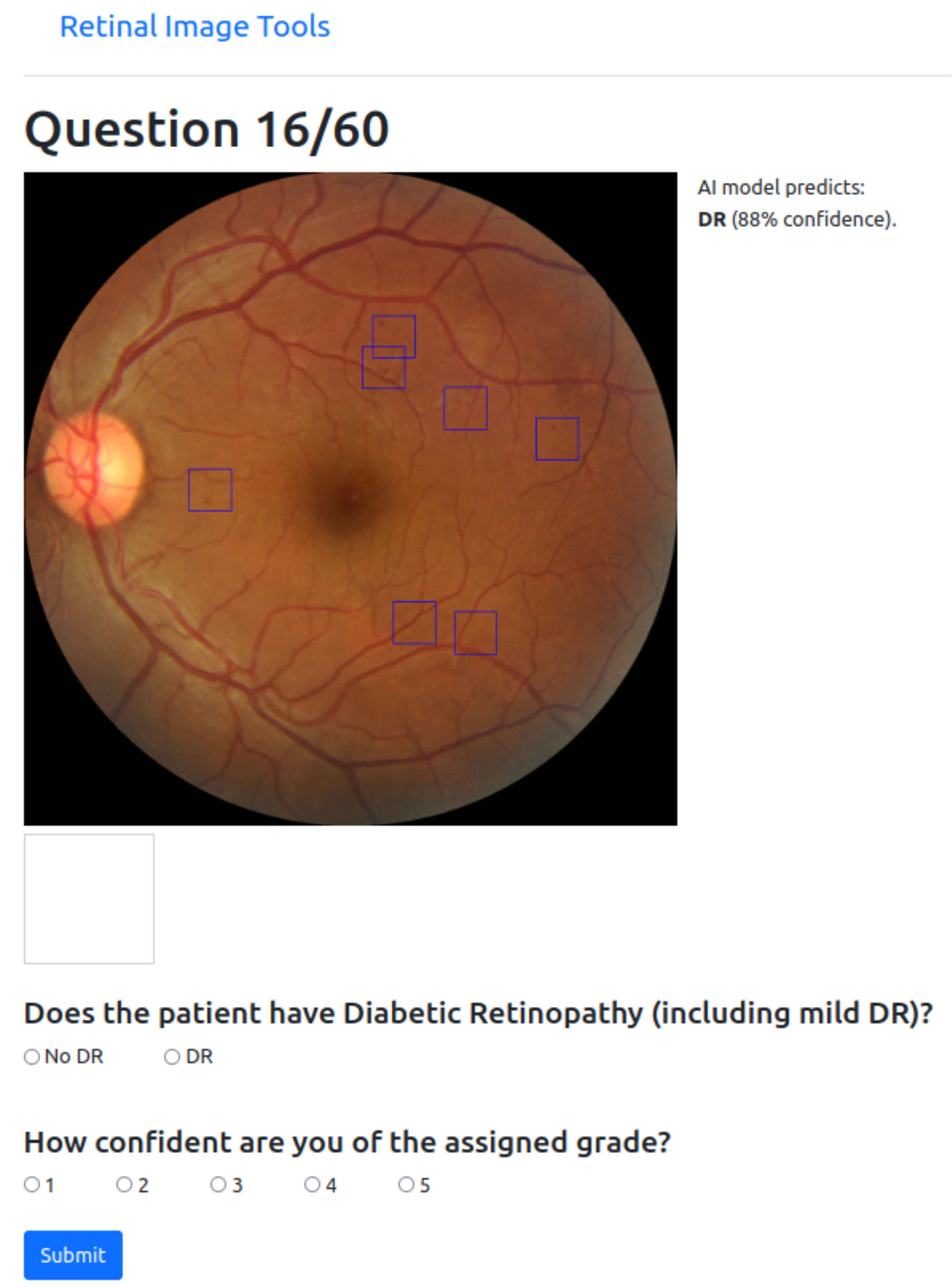

Supplement: S5 Fig — A fundus image is shown with the model’s prediction, its confidence level (from 0% to 100 %, with 100% being the highest confidence score), and explanation in the form of blue bounding boxes around the regions for which the AI model believes that they contain signs of DR. Based on this, the grader is asked to decide whether the corresponding patient has Diabetic Retinopathy (DR) of any severity, including mild DR. In addition, the grader is asked to rate the confidence of his/her decision on a scale from 1 (least confident) to 5 (most confident). By moving the mouse over a region of the image, an enlarged version of that region is displayed. The time taken to reach each decision (grading and confidence) is recorded. (TIF) [file pdig.0000831.s007.tif]

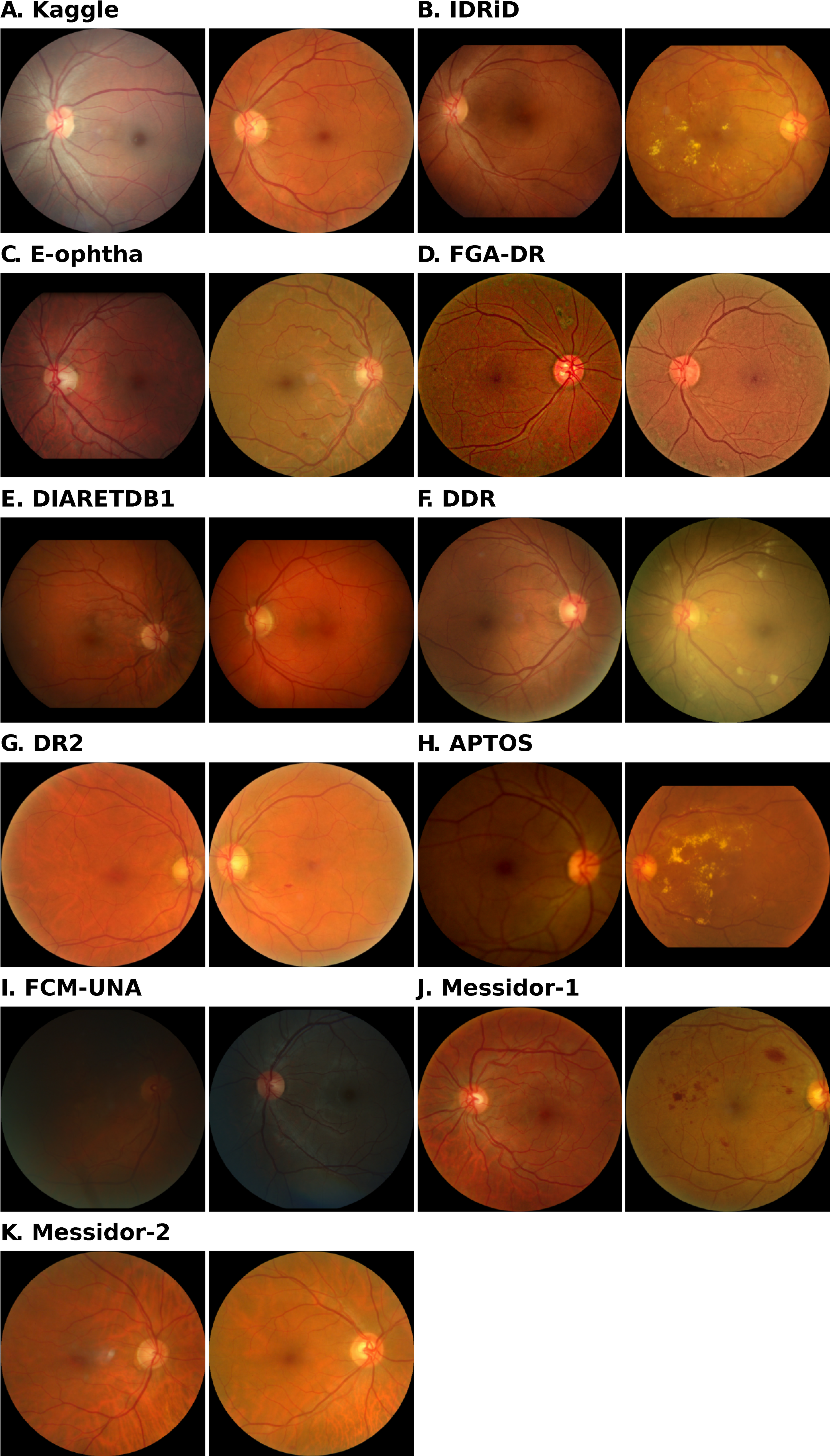

Supplement: S6 Fig — (TIF) [file pdig.0000831.s008.tif]

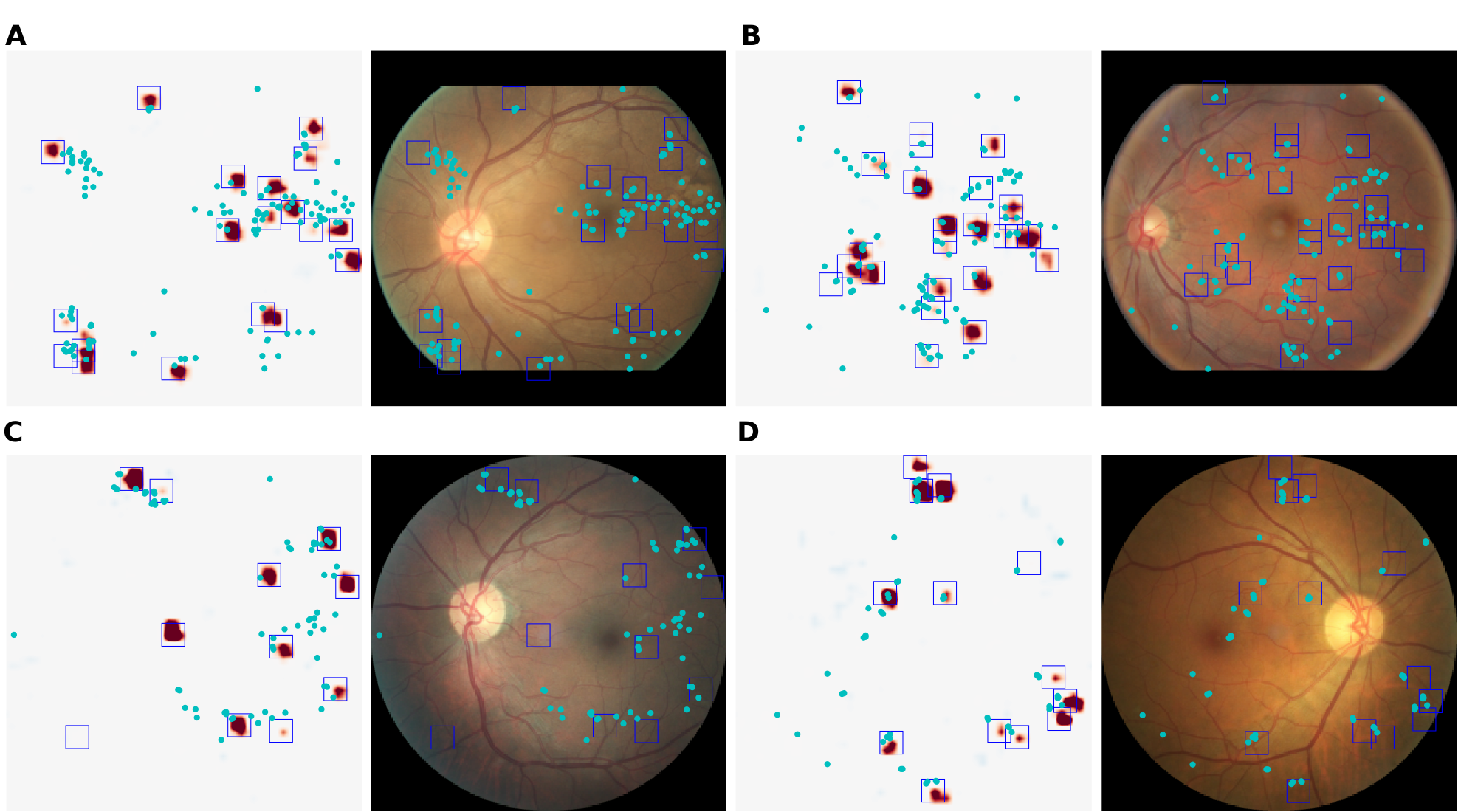

Supplement: S7 Fig — For each example, the left side shows the heatmap with clinicians’ annotations and bounding boxes around the regions of positive activation, while the right side shows the fundus image with clinicians’ annotations and bounding boxes around the regions of positive activation. (TIF) [file pdig.0000831.s009.tif]

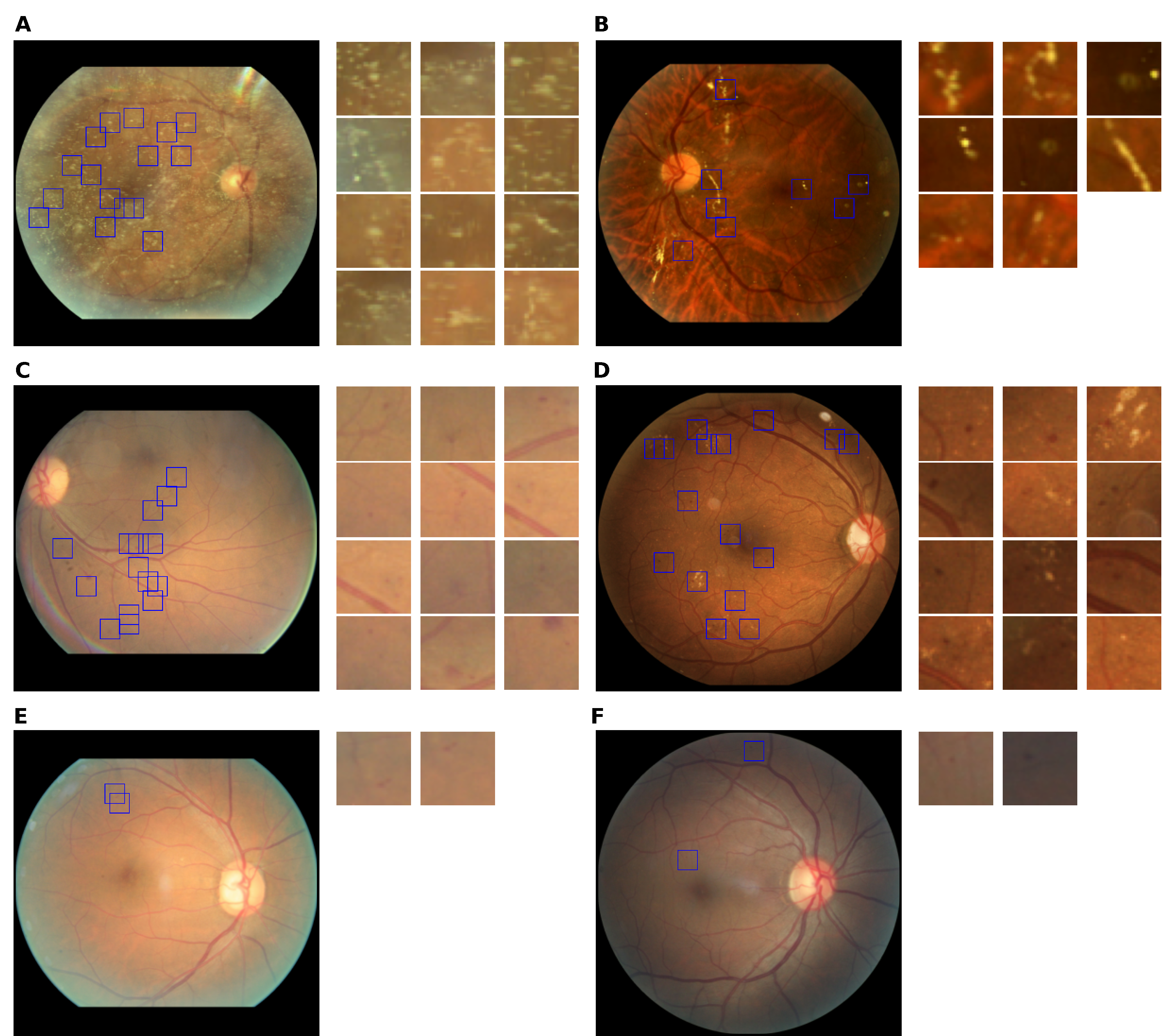

Supplement: S8 Fig — On the left side of each example, the image displays bounding boxes highlighting regions with positive activation. On the right side, the suspicious regions from the left are enlarged and arranged in descending order of evidence scores. (A) A false-positive image where the clinicians interpreted the suspicious regions as “vitreous opacities” and “uveitis vitreous cells,” respectively. (B) A false-positive image where one clinician identified the suspicious regions as “synchisis scintillans,” while the other suggested the patient may have recently received an intravitreal steroid injection. (C) A false-positive image where both clinicians identified the suspicious regions as “microaneurysms” possibly associated with bleeding. (D) A false-positive image where both clinicians recognized the suspicious regions as “microaneurysms” and “hard exudates”. (E, F) False-positive images where one clinician classified the image as DR while the other classifies it as no DR, citing the presence of only a single microaneurysm lesion in the suspicious regions. (TIF) [file pdig.0000831.s010.tif]

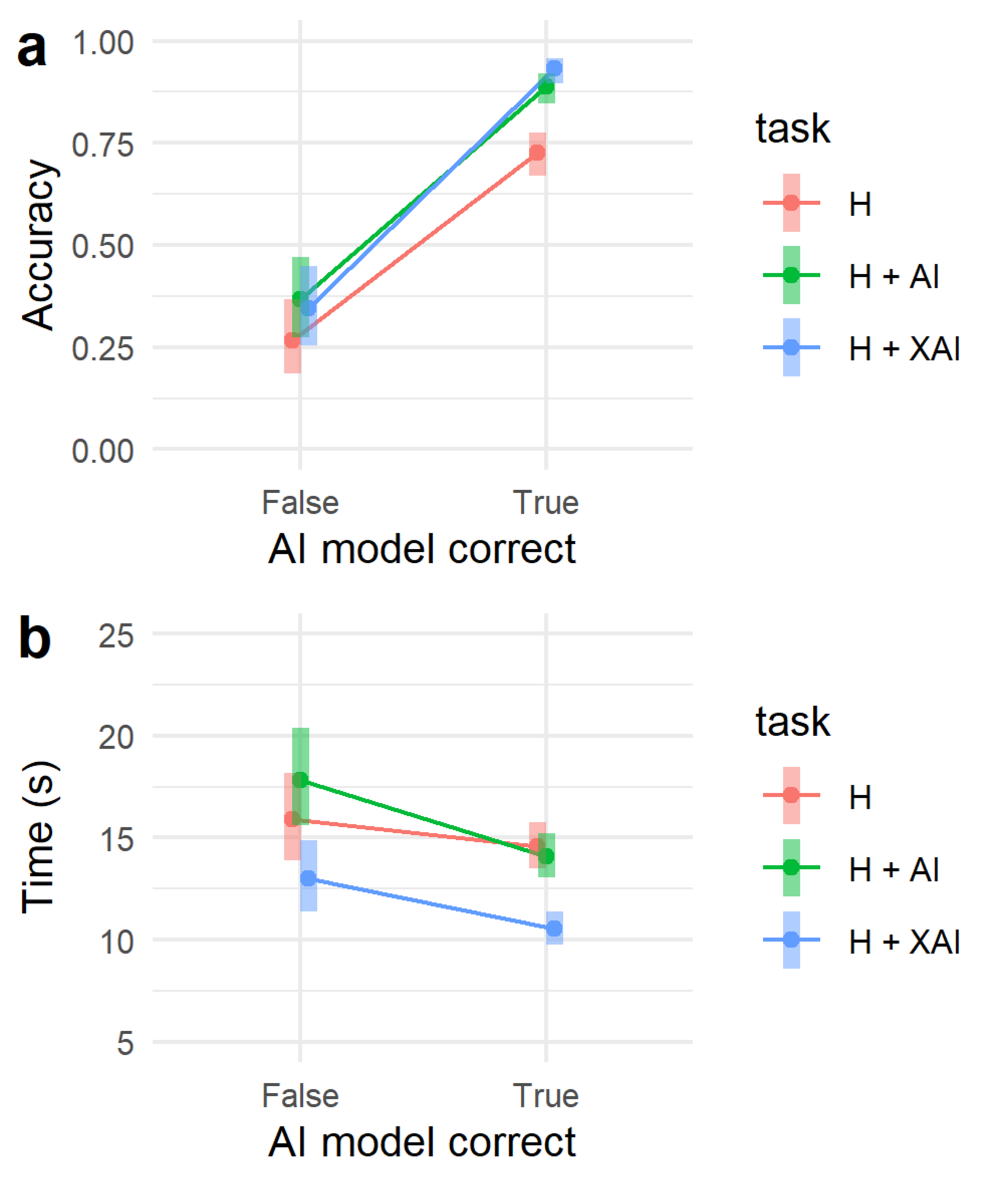

Supplement: S9 Fig — For all tasks, ophthalmologists’ accuracy is higher when the deep learning model makes the correct decision. For correct classifications, the AI assistance improves grading accuracy. For incorrect classification, it does not make it worse. (b) Ophthalmologists’ decision time decreases overall when the deep learning model makes the correct decision. When the AI model is correct, the explanation decreases decision time significantly, while it does not increase the decision time for incorrect decisions. (TIF) [file pdig.0000831.s011.tif]
