# Supplementary material for: An inherently interpretable AI model improves screening speed and accuracy for early diabetic retinopathy
Source: PLOS Digit Health. 2025 May 12;4(5):e0000831. doi: 10.1371/journal.pdig.0000831 (PMC12068651; doi:10.1371/journal.pdig.0000831)
Supplement: S1 Table — The precision of the model on each clinician annotation is calculated as the proportion of bounding boxes from regions highlighted on heatmaps containing lesions annotated by a grader. The random precision is obtained by drawing 20 random bounding boxes over each annotated image, excluding those falling in regions containing more than 10% black pixels. The union “∪” gives the precision of the model with the combined clinicians’ annotation masks, while the intersection “∩” gives the precision of the model with reference annotation masks obtained as the intersections of clinicians’ annotation over each image. (PDF) [file pdig.0000831.s002.pdf]

An inherently interpretable AI model improves screening speed and accuracy for early diabetic retinopathy

Supplementary Table 1

Djoumessi et al.

	Precision
Grader 1	0.709
Grader 2	0.610
Grader 3	0.923
Random	0.102
Grader 1 $\cup$ Grader 2 $\cup$ Grader 3	0.932
Grader 1 $\cap$ Grader 2 $\cap$ Grader 3	0.545

Supplementary Table 1. Summary of model performance on localizing DR-related lesions. The precision of the model on each clinician annotation is calculated as the proportion of bounding boxes from regions highlighted on heatmaps containing lesions annotated by a grader. The random precision is obtained by drawing 20 random bounding boxes over each annotated image, excluding those falling in regions containing more than 10% black pixels. The union "$\cup$" gives the precision of the model with the combined clinicians' annotation masks, while the intersection "$\cap$" gives the precision of the model with reference annotation masks obtained as the intersections of clinicians' annotation over each image.
