# Supplementary material for: An inherently interpretable AI model improves screening speed and accuracy for early diabetic retinopathy
Source: PLOS Digit Health. 2025 May 12;4(5):e0000831. doi: 10.1371/journal.pdig.0000831 (PMC12068651; doi:10.1371/journal.pdig.0000831)
Supplement: S2 Table — “Grader X - Grader Y” refers to the dice score between grader X and grader Y. The Dice score is calculated for each pair of graders as the overlap between their annotation using a patch size of 33×33 pixels corresponding to the receptive field of the model and considering different strides (s = 8, 32 for overlapping patches and s=33 for non-overlapping patches). “Grader X - Grader Y ∪ Grader Z” refers to the dice score between grader X, Y, and Z while “Grader Y ∪ Grader Z” is the union between grader Y and Z, and “Grader Y ∩ Grader Z” is the intersection between grader Y and Z. (PDF) [file pdig.0000831.s003.pdf]

An inherently interpretable AI model improves screening speed and accuracy for early diabetic retinopathy

Supplementary Table 2

Djoumessi et al.

	Dice (s=8)	Dice (s=32)	Dice (s=33)
Grader 1 - Grader 2	0.609	0.613	0.597
Grader 1 - Grader 3	0.545	0.543	0.542
Grader 2 - Grader 3	0.494	0.485	0.504
Grader 1 - Grader 2 ∪ Grader 3	0.546	0.551	0.544
Grader 2 - Grader 1 ∪ Grader 3	0.480	0.479	0.486
Grader 3 - Grader 1 ∪ Grader 2	0.613	0.613	0.616
Grader 1 - Grader 2 ∩ Grader 3	0.609	0.602	0.597
Grader 2 - Grader 1 ∩ Grader 3	0.480	0.479	0.486
Grader 3 - Grader 1 ∩ Grader 2	0.402	0.391	0.404

Supplementary Table 2. Inter-grader concordance on 65 fundus images. "Grader X - Grader Y" refers to the dice score between annotations of grader X and grader Y. The Dice score is calculated for each pair of graders as the overlap between their annotation using a patch size of 33×33 pixels corresponding to the receptive field of the model and considering different strides (s = 8, 32 for overlapping patches and s=33 for non-overlapping patches). "Grader X - Grader Y ∪ Grader Z" refers to the dice score between grader X, Y, and Z while "Grader Y ∪ Grader Z" is the union between grader Y and Z, and "Grader Y ∩ Grader Z" is the intersection between grader Y and Z.
