# Supplementary material for: An inherently interpretable AI model improves screening speed and accuracy for early diabetic retinopathy
Source: PLOS Digit Health. 2025 May 12;4(5):e0000831. doi: 10.1371/journal.pdig.0000831 (PMC12068651; doi:10.1371/journal.pdig.0000831)
Supplement: Attachment — Submitted filename: Comprehensive Manuscript Review for PLOS Digital Health PLOS.pdf [file pdig.0000831.s012.pdf]

Comprehensive Manuscript Review for PLOS Digital Health

Title: An Inherently Interpretable AI Model Improves Speed and Accuracy of Screening for Early Diabetic Retinopathy

1. Significance and Innovation

This study presents a significant advancement in the application of artificial intelligence to medical imaging, specifically in the context of diabetic retinopathy (DR) screening. The innovative aspect lies in the development of an inherently interpretable deep learning model that maintains high performance while providing transparent decision-making processes. This approach addresses a critical gap in current AI systems for medical diagnosis, which often operate as "black boxes."

The significance of this work extends beyond the specific application to DR screening. It demonstrates a pathway for developing AI systems that can be more readily integrated into clinical workflows, potentially increasing trust and adoption among healthcare professionals. This aligns with the growing emphasis on explainable AI in healthcare, addressing concerns about transparency and accountability in AI-assisted medical decision-making.

2. Methodological Rigor and Data Quality

The study's methodology demonstrates a high level of rigor, which strengthens the validity of its findings:

a) Data Diversity: The use of eleven public datasets of fundus images, including international sources, enhances the model's generalizability. This diverse data foundation is crucial for developing robust AI systems that can perform consistently across different populations and clinical settings.

b) Model Architecture: The choice of a Sparse BagNet model is well-justified. This architecture allows for the extraction of local evidence in an inherently interpretable manner, which is a key innovation of the study. The comparison with a standard ResNet-50 model provides a valuable benchmark against current state-of-the-art approaches.

c) Evaluation Metrics: The comprehensive set of evaluation metrics (AUC, sensitivity, specificity, and accuracy) provides a thorough assessment of the model's performance. The inclusion of clinician validation for the quality of explanations adds a crucial practical dimension to the evaluation.

d) Statistical Analysis: The use of confidence intervals for key metrics adds robustness to the findings, allowing for a more nuanced interpretation of the results.

3. Results and Clinical Implications

The study's results are compelling and have significant clinical implications:

a) Performance Parity: The interpretable model achieving performance comparable to "black box" models is a crucial finding. It demonstrates that transparency need not come at the cost of accuracy, a common concern in the development of explainable AI systems.

b) Improved Diagnostic Accuracy: The 17.5% improvement in diagnostic accuracy for mild DR is particularly noteworthy. Early detection of DR is critical for preventing vision loss, and this improvement could translate to better patient outcomes.

c) Reduced Screening Time: The 25% reduction in screening time is a significant practical advantage. In resource-constrained healthcare settings, this could lead to increased screening capacity and potentially earlier interventions for at-risk patients.

d) Enhanced Human-AI Collaboration: The ability of the model to provide clinically relevant evidence maps could fundamentally change how clinicians interact with AI systems. This has the potential to create a more synergistic relationship between AI and human expertise in medical diagnosis.

4. Limitations and Future Directions

While the study is robust, there are areas where further research could enhance its impact:

a) Real-world Validation: Prospective studies in diverse clinical settings would provide valuable insights into the model's performance and utility in real-world scenarios.

b) Generalizability: Further exploration of the model's performance across different ethnic groups and varying image qualities would strengthen its applicability in global healthcare settings.

c) Long-term Impact: Studies on how the use of such interpretable models affects clinical decision-making processes and patient outcomes over time would be valuable.

d) Integration Challenges: Research into the practical aspects of integrating such systems into existing clinical workflows and electronic health record systems would be beneficial.

5. Ethical Considerations

The study appropriately addresses ethical concerns by emphasizing transparency and interpretability. However, future work might benefit from a more in-depth discussion of potential biases in AI systems and strategies to mitigate them.

6. Presentation and Accessibility

The manuscript is well-structured and clearly written, making it accessible to a broad audience of clinicians, AI researchers, and healthcare policymakers. The inclusion of code and data availability enhances reproducibility, which is crucial for building trust in AI research.

Conclusion and Recommendation

This manuscript represents a significant contribution to the field of AI in healthcare. It not only advances the technical aspects of interpretable AI but also addresses crucial practical and ethical considerations in medical AI applications. The study's findings have the potential to influence the development of future AI systems in healthcare, promoting a more transparent and collaborative approach to AI-assisted diagnosis.

Given its innovative approach, robust methodology, and significant clinical implications, I strongly recommend this article for publication in PLOS Digital Health. It aligns well with the journal's scope and could serve as a landmark paper in the evolving landscape of interpretable AI in medicine.

This work opens up exciting avenues for future research and could potentially influence regulatory approaches to AI in healthcare, emphasizing the importance of interpretability alongside performance metrics. As such, it deserves wide dissemination and discussion within the scientific and medical communities.
