# Supplementary material for: An inherently interpretable AI model improves screening speed and accuracy for early diabetic retinopathy
Source: PLOS Digit Health. 2025 May 12;4(5):e0000831. doi: 10.1371/journal.pdig.0000831 (PMC12068651; doi:10.1371/journal.pdig.0000831)
Supplement: Attachment — Submitted filename: Djoumessi et al. Rebuttal .pdf [file pdig.0000831.s013.pdf]

Reviewer #1:

This manuscript presents an innovative approach to diabetic retinopathy screening using interpretable artificial intelligence. The authors have developed a sparse BagNet model that achieves strong performance while providing transparent explanations for its decisions. After careful review, I find several key strengths and areas for improvement.
The study's methodological approach demonstrates commendable rigor. The authors trained their model on a substantial dataset of 34,350 fundus images and validated it across ten external datasets, providing robust evidence for generalizability. The inclusion of a retrospective reader study with six ophthalmologists adds particular value, as it demonstrates real-world clinical utility. The reported improvements in diagnostic accuracy (17.5% for challenging cases) and screening efficiency (25% reduction in time) are both statistically and clinically significant.
The technical innovation of the sparse BagNet architecture represents an important advance in interpretable AI for medical imaging. Unlike post-hoc explanation methods that attempt to rationalize decisions after the fact, this approach builds interpretability directly into the model's architecture. The achievement of 0.906 accuracy while maintaining interpretability demonstrates that transparency need not come at the cost of performance.

We thank the reviewer for their positive review, clearly highlighting the strengths of our approach.

However, several aspects of the methodology require clarification or enhancement:
The manuscript would benefit from a more detailed explanation of the hyperparameter selection process, particularly regarding the sparsity parameter λ. Understanding how this parameter was optimized would help readers implement similar approaches.

We added a brief additional description of how the sparsity was chosen to the Results – in this case, simply heuristically as the point before accuracy started to drop more strongly, as we already pointed out in the Methods (see Supp. Fig. 2).

Additionally, the EfficientNet-based quality filtering process needs more detailed documentation to ensure reproducibility.

We have added details of the quality filtering process. Since finishing the paper, we have further developed our quality filtering models and made them available as part of the Fundus Image Toolbox (https://github.com/berenslab/fundus_image_toolbox).

The clinical implementation section would be strengthened by addressing practical considerations such as hardware requirements, expected processing times in real-world settings, and integration guidelines for existing clinical workflows. These details are crucial for healthcare institutions considering adoption of the technology.

We thank the reviewer for bringing this up – we added a section to the Discussion regarding integration into clinical workflows and provide some information about technical requirements as well.

For the validation study, the authors should provide more detail about the criteria used to select the 65 images for expert annotation. Including inter-rater reliability metrics would help readers assess the robustness of these annotations as a ground truth reference.

As described in Section 2.1, the images were randomly chosen for annotation, 20 from grade 1 and 45 from grade 2. Intergrader reliability metrics were also already included in the original submission (Supp. Table 2).

The statistical analysis, while generally sound, would benefit from the addition of:

Power analysis for the reader study
Effect size calculations for key comparisons
Confidence intervals for timing measurements

We agree with the reviewers that all of these are important aspects. (1) We did not perform a formal power calculation for the reader study, but rather tried to recruit as many colleagues from the ophthalmology department as we could. We state this now more clearly in the manuscript. (2) For clarity of the manuscript, we did not include effect sizes in addition to all the statistical information already provided. They can be easily computed using the provided R code. (3) CIs for timing measurements were included both in Fig. 5 and in the text on page 7.

I also recommend expanding the discussion of limitations to address:

Potential biases in the training dataset
Generalizability across different patient demographics
Performance variations across different imaging equipment
Long-term validation requirements

We again agree with the reviewer that these are important aspects – we expanded our Methods and Discussion accordingly.

The authors should consider adding technical comparisons with other interpretable architectures beyond standard DNNs. This would help readers understand the relative advantages and trade-offs of the sparse BagNet approach.

We thank the author for bringing this up – we included comparisons to the most relevant competitors, namely post-hoc methods from standard DNNs in Fig. 4. We are not aware of any inherently interpretable architecture that would be applicable to this setting in a straightforward manner. We included a brief discussion of alternatives such as Prototype Networks in the Discussion.

Despite these suggestions for improvement, the manuscript represents a significant contribution to the field. The combination of technical innovation, thorough validation, and demonstrated clinical benefit makes it suitable for publication in PLOS Digital Health. The recommended revisions would enhance what is already a valuable addition to the literature on AI in healthcare.
Regarding ethical considerations, the study appropriately obtained institutional review board approval (Ref No. 249/2023BO2) and handled patient data appropriately. The authors have made their implementation and study data available through GitHub, supporting reproducibility and further research.
In conclusion, I recommend acceptance with minor revisions. This work advances both the

technical and clinical aspects of AI-assisted diabetic retinopathy screening, with particular strength in addressing the critical need for interpretability in clinical AI systems.

Again, we thank the reviewer for their positive feedback.

Reviewer #2: This manuscript presents a well-executed study on interpretable AI for diabetic retinopathy screening. The methodology is rigorous, with comprehensive validation and a strong reader study demonstrating clinical utility. While the statistical analysis and results are compelling, particularly regarding reduced screening time without accuracy loss, some minor revisions are needed. The data availability statement needs expansion with specific repository links, and the discussion of clinical implementation could be strengthened. The writing is clear with only minor grammatical issues to address. Overall, this is valuable work that, with these minor revisions, will be suitable for publication in PLOS Digital Health.

We thank the reviewer for their positive feedback – the data sharing statement in the PLoS form was adjusted to match that in the paper. We also added a clinical implementation paragraph to the discussion.
